# Foreign Bodies in the Rectum

**Published:** 1886-05

**Authors:** W. N. Perkins

**Affiliations:** Sabine Pass, Texas


### FOREIGN BODIES IN THE RECTUM.

*By W. N. Perkins, M. D., Sabine Pass.*

(For Daniel's Texas Medical Journal.)

I OFFER a brief report of two cases from memorandum book, of foreign bodies in the rectum, which you can publish if you think they are of sufficient interest.

Such cases are not very common, and I am of the opinion that they are sometimes not recognized, but treated as sporadic dysentery, with one dose of medicine after another, when the speculum, finger or forceps would have been the proper remedies. The subjects of both cases were robust and well developed negro men.

First case—A man weighing about 160 pounds, 54 years of age, with no deficiency of physical organization, except a hare lip, and cleft palate, on account of which his articulation was so imperfect that it was difficult to understand him. He had been suffering severely for a day and night. I heard his cries before entering his house. When I entered his room, he was over the chamber, and the impress of pain was as vividly stamped upon his countenance as I ever saw it upon a human being. He exclaimed: "Oh, doctor, for God's sake do something for me!" I immediately administered a dose of morphine hypodermically, and though a large dose, it gave him only partial relief. The excessive tenesmus and tormina caused me to believe that there was a foreign substance in the rectum of some kind. Having placed him in a proper position, I introduced my finger, and to my great astonishment, discovered a large needle, such as is used for sewing quilts in frames, lying obliquely across the rectum, just above the sphincter, and firmly imbedded in its walls. The needle had a double thread in it, six or eight inches long. I succeeded with difficulty in extracting it; after which he quickly recovered. His wife recognized the needle, and remarked that she knew it was lost and had more than once hunted for it, but had never thought to look for it where it was found.

Second case—An unusually robust and well developed young negro man, 28 years old. I treated him for dysentery. When first called to him he was having bloody stools with much tenesmus. I prescribed sulphate of magnesia and tr. opii. At my second visit he had had several copious evacuations with but slight temporary relief. I examined his rectum and extracted a bone, the vertebra of a hog, as large as a small sized hen egg. It was of a porous worm eaten nature, from the appearance of which, I supposed it had been in some part of the alimentary canal for several weeks. He recollected having made his dinner on back-bone about three weeks before his attack of sickness.

